# Fever screening during the influenza (H1N1-2009) pandemic at Narita International Airport, Japan

**DOI:** 10.1186/1471-2334-11-111

**Published:** 2011-05-03

**Authors:** Hiroshi Nishiura, Kazuko Kamiya

**Affiliations:** 1 PRESTO, Japan Science and Technology Agency, 4-1-8 Honcho, Kawaguchi, Saitama 332-0012, Japan; 2 Theoretical Epidemiology, University of Utrecht, Yalelaan 7, Utrecht, 3584 CL, The Netherlands; 3 School of Public Health, Li Ka Shing Faculty of Medicine, The University of Hong Kong, Pokfulam, Hong Kong Special Administrative Region, China; 4 Narita Airport Quarantine Station, New Tokyo International Airport, Passenger Terminal 2, Narita, Chiba 282-0004, Japan

## Abstract

**Background:**

Entry screening tends to start with a search for febrile international passengers, and infrared thermoscanners have been employed for fever screening in Japan. We aimed to retrospectively assess the feasibility of detecting influenza cases based on fever screening as a sole measure.

**Methods:**

Two datasets were collected at Narita International Airport during the 2009 pandemic. The first contained confirmed influenza cases (n = 16) whose diagnosis took place at the airport during the early stages of the pandemic, and the second contained a selected and suspected fraction of passengers (self-reported or detected by an infrared thermoscanner; n = 1,049) screened from September 2009 to January 2010. The sensitivity of fever (38.0°C) for detecting H1N1-2009 was estimated, and the diagnostic performances of the infrared thermoscanners in detecting hyperthermia at cut-off levels of 37.5°C, 38.0°C and 38.5°C were also estimated.

**Results:**

The sensitivity of fever for detecting H1N1-2009 cases upon arrival was estimated to be 22.2% (95% confidence interval: 0, 55.6) among nine confirmed H1N1-2009 cases, and 55.6% of the H1N1-2009 cases were under antipyretic medications upon arrival. The sensitivity and specificity of the infrared thermoscanners in detecting hyperthermia ranged from 50.8-70.4% and 63.6-81.7%, respectively. The positive predictive value appeared to be as low as 37.3-68.0%.

**Conclusions:**

The sensitivity of entry screening is a product of the sensitivity of fever for detecting influenza cases and the sensitivity of the infrared thermoscanners in detecting fever. Given the additional presence of confounding factors and unrestricted medications among passengers, reliance on fever alone is unlikely to be feasible as an entry screening measure.

## Background

The rapid international spread of severe acute respiratory syndrome (SARS) from 2002 to 2003 led countries around the world to extensively assess the entry screening measures at their international borders as one of the countermeasures to prevent the global spread of infectious diseases [1,2]. Pandemic influenza has been one of the most important subjects for entry screening [3]. Including an analysis of the historical records of maritime quarantine during the 1918-1919 influenza pandemic [4], many scientific discussions concerning the scientific value and public health performance of entry screening took place prior to the 2009 pandemic. Although the efficacy of entry screening in correctly detecting and diagnosing influenza cases is likely to be small, mainly because of the impossibility of detecting incubating individuals at the border [5,6] and the presence of asymptomatic cases [7-9], many countries adopted entry screening measures to some extent during the early stages of the 2009 pandemic [10]. Japan followed its original guideline [11] to enforce entry screening at international airports as well as other border control measures during the very early stages of the 2009 pandemic, with the aims of detecting influenza cases at the border and preventing secondary transmissions arising from potentially exposed individuals through strict quarantine (e.g. at hotels close to airports) or voluntary home quarantine.

Since the diagnostic criteria and definitions of both SARS and influenza-like illness include fever, entry screening tends to start with a search for febrile international passengers, and such fever screening has tended to largely rely on the use of infrared thermoscanners because of their non-invasive nature and the need to screen massive numbers of travelers at the border [12-14]. Because of the relatively high sensitivity and specificity, the negative predictive value (NPV) of infrared thermoscanners in excluding non-febrile passengers is believed to be high [15-19], which supports the use of infrared thermoscanners for releasing negative individuals (i.e. a strict screening measure through diagnosis by exclusion), under an important assumption that the prevalence of infected individuals is small among the total number of passengers and with the expectation that "cases" are represented as febrile passengers. Although border control does not fully rely on infrared thermoscanners to detect febrile passengers, Narita International Airport (also known as Tokyo-Narita Airport or New Tokyo International Airport), comprising the largest international airport in Japan and dealing with 58% of arriving international passengers, has placed stationary infrared thermoscanners as an aid to monitor and screen for fever among arriving international passengers since 2003. However, despite the high diagnostic accuracy and NPV under the above-mentioned assumption and expectation, the readings of infrared thermoscanners are known to be influenced by several confounding factors including age and outdoor temperature, and the small positive predictive value (PPV) with the small prevalence of febrile passengers is not believed to realistically permit less strict entry screening (e.g. use of infrared thermoscanners to actively detect hyperthermia) [20-22]. The validity of fever screening in relation to its theoretical rationale (e.g. the above-mentioned assumption and expectation) should be assessed in practical settings.

Japan is one of the countries that implemented the most strict entry screening during the early stages of the 2009 pandemic [10]. This allowed us to retrospectively analyze epidemiological datasets of confirmed cases whose diagnosis took place at an international border during the early entry screening practice and of a portion of passengers screened by the infrared thermoscanners. The datasets of the influenza cases and passengers provide us with a unique opportunity to critically investigate the public health performance of fever screening in correctly detecting and diagnosing influenza (H1N1-2009) at international borders. The purpose of the present study was to retrospectively assess the feasibility of detecting influenza cases based on fever screening as a sole measure through the analysis of actual entry screening data, thereby identifying practical issues surrounding fever screening of passengers including influenza cases.

## Methods

**Figure 1:**
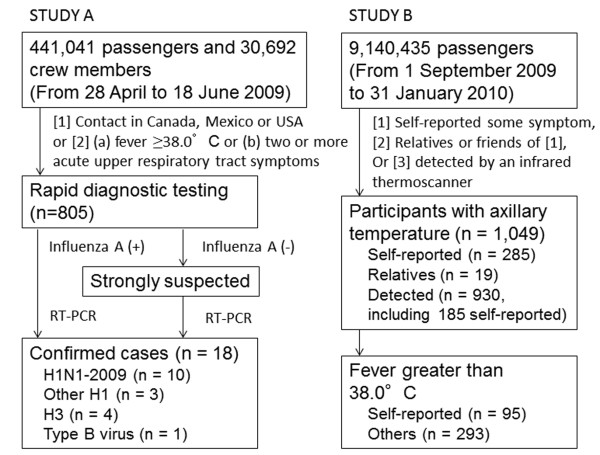
**Flow chart of participants in the study**. Two datasets were collected at Narita International Airport. The first contained confirmed influenza cases whose diagnosis took place at the airport during the early stages of the pandemic (Study A). The second contained a selected and suspected fraction of passengers (self-reported or detected by an infrared thermoscanner) screened from September 2009 to January 2010 (Study B).

In the present study, we analyzed two different datasets collected at Narita International Airport, which receives approximately 87,000 international flights per year (i.e. 240 flights per day) and through which approximately 18 million passengers per year enter Japan (i.e. 40,000-50,000 passengers per day) including Japanese passengers returning from abroad. The first dataset contained the limited number of confirmed cases infected with H1N1-2009 or other influenza viruses whose diagnosis took place at the airport during the very early stages of the 2009 pandemic, and the second dataset contained non-randomly sampled passengers, comprising a selected and suspected fraction of passengers (self-reported or detected by an infrared thermoscanner) arriving at Narita International Airport from September 2009 to January 2010 (Figure 1).

### Confirmed cases

The first dataset was based on strict entry screening measures implemented from 28 April to 18 June 2009, which targeted passengers arriving from Canada, Mexico and the United States. The strictest border control measures (i.e. those involving fever screening of passengers on board before disembarkation from an arriving aircraft) were performed until 21 May. From 22 May to 18 June, a clinical examination and laboratory testing were performed for self-reporting passengers and those detected by the stationary infrared thermoscanners. Passengers with a travel history to the above-mentioned three countries with fever greater than 38.0°C (directly measured by the tympanic or axillary temperature, for example) or with two or more acute upper respiratory tract symptoms (e.g. cough, sputum or breathlessness) underwent rapid diagnostic testing for influenza. Briefly, nasal swab specimens were taken for the rapid diagnostic testing and, if positive for influenza type A virus, a confirmatory diagnosis was made by RT-PCR. The primers for real-time RT-PCR for H1N1-2009 detection were developed by the National Institute of Infectious Diseases and became available on 29 April 2009 [23]. During the 52-day screening period, a total of 1,903 commercial aircrafts landed at Narita International Airport from the three countries bringing 441,041 passengers and 30,692 airline crew members [24]. Among these, 805 persons underwent rapid diagnostic testing, and 15 tested positive. Including those who tested negative but were strongly suspected of having the disease (e.g. those with a history of apparent contact with a known case), a total of 18 cases were confirmed as having influenza (Figure 1). Among these cases, 10 had H1N1-2009, 7 had other influenza type A virus subtypes (four with H3 and three with H1 other than H1N1-2009) and one had influenza type B virus. One of the 10 H1N1-2009 cases was healthy upon arrival, but had a history of contact with other symptomatic cases. Since this case developed the illness during quarantine at a hotel, the case was excluded from our analysis. The temperature of one H3 case upon arrival was unknown. Accordingly, a total of 9 cases with H1N1-2009 and 7 cases with other influenza viruses with data regarding age, gender, history of medications prior to arrival and axillary temperature upon arrival were evaluated.

### Screened passengers

The other dataset included data for axillary temperature, surface temperature measured by an infrared thermoscanner and other variables among a total of 1,049 passengers arriving at Narita International Airport from 1 September 2009 to 31 January 2010. During the 5-month study period, a total of 9,140,435 passengers entered Japan through Narita International Airport, and all were screened by infrared thermoscanners. A total of eight TVS-500 infrared thermoscanners (NEC/AVIO Infrared Technologies Co. Ltd., Tokyo, Japan) were placed near the quarantine station before immigration. The infrared sensors optically scanned the surface of each passenger, and the temperature distributions were recorded as two-dimensional thermal images. Our subjects comprised a selected and suspected fraction of passengers among the total passengers, who fulfilled one of the following selection criteria: (a) those who self-reported some symptom or actively visited the health consultation room of the quarantine station; (b) relatives or friends of self-reporting individuals; or (c) those who were detected by an infrared thermoscanner (based on a predefined threshold reading of 35.4°C) and were asked by quarantine officers to undergo further examinations. Hereafter, we refer to these 1,049 passengers as the "selected and suspected fraction" of passengers, because the passengers were selected based on the above-mentioned criteria and were more likely to be suspected of fever than the remaining passengers.

**Figure 2:**
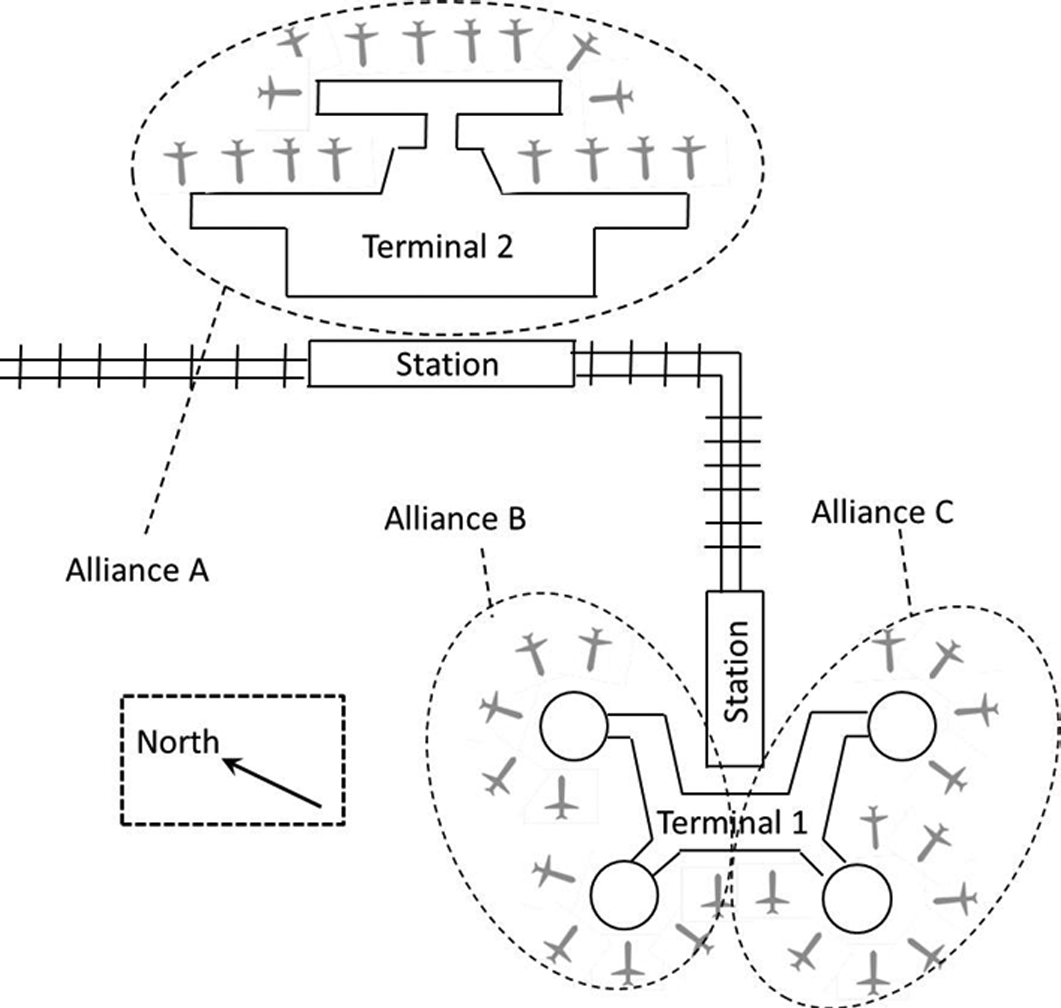
**Simplified map of Narita International Airport**. The airport has two discrete terminals. A total of four infrared thermoscanners were placed in each terminal. Terminal 2 is mostly used by alliance A, while Terminal 1 is roughly divided into two groups of satellites used by alliances B and C, respectively. The stationary infrared thermoscanners were set up near the quarantine station before immigration.

Figure 2 shows a map of Narita International Airport, which employs a satellite terminal design (i.e. an airport building detached from other airport buildings so that aircraft can park around its entire circumference). There are two terminals, namely Terminal 1 with four satellites and Terminal 2 with two satellites. These satellites can be crudely classified into three areas, and each is utilized by a single alliance of airline companies. Four infrared thermoscanners were set up in each terminal. The distances between the infrared thermoscanners and the passengers varied slightly in the satellites, being 4-19 m in Terminal 1 and 3-10 m in Terminal 2.

Guided by quarantine officers, all the subjects voluntarily entered the health consultation room. Upon entrance, the axillary temperature of the subjects was measured once using a C202 axillary thermometer (Termo Corporation, Tokyo, Japan). The sensor was directly inserted into the axilla, and the temperature was measured by a thermistor. The temperature was displayed at approximately 90 seconds after the insertion. In addition to the temperature, we collected information regarding age, gender, aircraft (i.e. place of embarkation), self-reporting (i.e. presence or absence of voluntary reporting of any symptoms) and information of the satellite where the surface temperature was measured by an infrared thermoscanner (i.e. alliances A, B or C). Since air-conditioning greatly influences room temperature variations within the airport, and because the room temperature also varies with arrival gates and satellites (e.g. depending on individual routes of entry), we were not able to measure the room temperature for each subject. History of medications prior to the screening was not collected systematically.

### Statistical analysis

For both datasets, we used the axillary temperature as a reference variable, and axillary temperatures above defined thresholds were considered to be hyperthermia (our outcome variable). First, we documented the summary statistics of the demographic variables and axillary temperatures for both datasets. Second, using the first dataset, we examined the sensitivity of fever for detecting influenza among the sample of confirmed cases, by using three different cut-off levels for defining hyperthermia (37.5°C, 38.0°C and 38.5°C) based on the axillary temperature upon arrival. Because the sample size was small, we computed the exact 95% confidence interval (CI) of the sensitivity, using the quantile function of the binomial distribution. We also examined the associations between hyperthermia and types of influenza virus (H1N1-2009 or not), age, gender and history of medications.

Third, among the 1,049 screened passengers, we measured the associations of hyperthermia with age, gender, place of embarkation (categorized into six regions of the World Health Organization, but grouping Southeast-Asia and Western Pacific regions into one region owing to their geographic closeness), self-reporting (dichotomous) and satellite of measurement (categorized by three areas as shown in Figure 2). Except for the axillary temperature and the surface temperature measured by an infrared thermoscanner, only age was a continuous variable. We employed the Welch test to examine the association between hyperthermia and age. For all the remaining variables, we used Fisher's exact test or the *χ*^2 ^test.

Fourth, we assessed the univariate correlation and association between the axillary temperature (outcome variable) and the surface temperature measured by an infrared thermoscanner. Pearson's product-moment correlation was employed to examine the correlation between two continuous variables. Subsequently, the diagnostic performances (including sensitivity, specificity and area under the receiver operating characteristic curve (AUC)) of the infrared thermoscanners were estimated along with the PPV and NPV. We employed the Youden index (i.e. sensitivity plus specificity minus 1) to identify the sensitivity and specificity of the infrared thermoscanners at an optimal threshold of the surface temperature. The 95% CIs of the sensitivity and specificity were computed using normal approximation to the binomial distribution, while the calculations of the 95% CIs of the PPV and NPV were based on the Wald method with the PPV and NPV variances determined by the delta method [25]. For calculation of the 95% CI of the AUC, we employed the Wald method using logit transformation of the AUC [26]. Lastly, we measured an adjusted AUC by incorporating a demographic variable that appeared to be a potential confounding factor of hyperthermia (i.e. age) by employing a multiple logistic regression. Since our selection criterion (c) for the 1,049 passengers already included those detected by the thermoscanners, we also assessed the above-mentioned diagnostic performances of the thermoscanners in identifying fever among the sample of self-reporting passengers only.

### Ethical considerations

The study conformed to the principles of the Helsinki Declaration. Eligible subjects were voluntarily enrolled, and informed consent was obtained before the enrollment. The survey was conducted during the entry screening practice following the guideline of the Japan Pandemic Influenza Plan issued by the Government of Japan [11]. The fever screening, health examination and laboratory testing were conducted according to the Quarantine Act (Articles 12 and 13), and the use of the infrared thermoscanners and examination of axillary temperature adhered to the Health Service Bureau Notice issued by the Tuberculosis and Infectious Disease Control Division of the Ministry of Health, Labour and Welfare of Japan. The analysis of the data and its publication are permitted by Article 27-2 of the Quarantine Act. No names (only ID numbers) were assigned to each study participant and the data were anonymously analyzed.

## Results

### Fever among confirmed cases (n = 16)

The mean (standard deviation (SD)) age of all the confirmed cases was 30.5 (16.4) years. The ages did not differ significantly between patients with H1N1-2009 and the other influenza viruses (p = 0.11). Males accounted for 9 cases (56.3%), and gender was not significantly associated with H1N1-2009 (p = 0.13). A total of 13 cases (81.3%) were under medications upon arrival. Five of the 9 H1N1-2009 cases (55.6%) had taken commercially available cold/cough medications containing antipyretic substances, and one child case among the remaining four cases took an antibiotic (azithromycin) prior to arrival. These medications were started at 20 hours to 2 days before arrival. All 7 cases with the other influenza viruses were under medications: five with commercially available cold/cough medications containing antipyretic substances, one with oseltamivir and one with an antibiotic (cefcapene pivoxil hydrochloride). Medications were not significantly associated with H1N1-2009, when the antibiotics were both included and excluded (p = 0.21 and p = 0.31, respectively).

**Figure 3:**
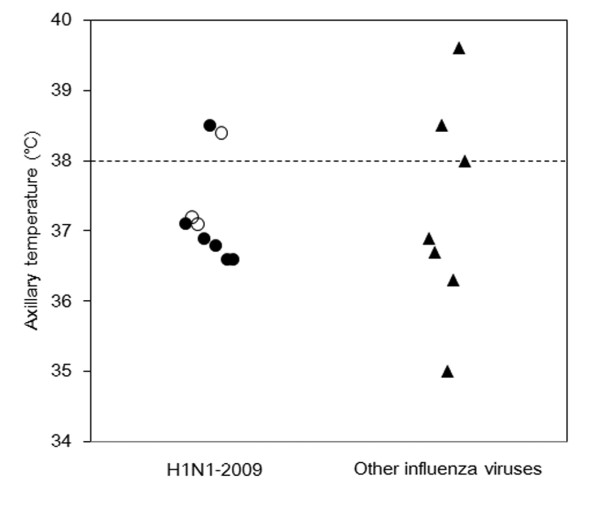
**Distribution of the axillary temperatures among the confirmed influenza cases**. The axillary temperatures upon arrival were compared between the cases with H1N1-2009 (n = 9) and the cases with other influenza viruses (n = 7). The confirmed cases represent patients whose diagnosis took place at Narita International Airport from 28 April to 18 June 2009. Unfilled symbols represent passengers without medications upon arrival and filled symbols represent passengers with medications. The horizontal dashed line is the reference line of 38.0°C, above which cases may be regarded as having hyperthermia.

Among the 9 confirmed cases with H1N1-2009, the axillary temperature upon arrival ranged from 36.6-38.5°C with a mean (SD) of 37.2°C (0.7°C). The axillary temperature of the cases with the other influenza viruses ranged from 35.0-39.6°C with a mean (SD) of 37.3°C (1.5°C). The axillary temperature did not differ significantly between the two groups (p = 0.95; Figure 3), and the proportions of hyperthermia also did not differ significantly between the two groups for the cut-off levels of 37.5°C, 38.0°C and 38.5°C (p > 0.05 for all cut-off levels). For the cut-off levels of both 37.5°C and 38.0°C, the sensitivities of hyperthermia for detecting influenza were estimated to be 22.2% (95% CI: 0, 56.0) for H1N1-2009 and 42.9% (95% CI: 14.3, 85.7) for the other influenza viruses. Using 38.5°C as the cut-off level, the sensitivities were estimated to be 11.1% (95% CI: 0, 33.3) for H1N1-2009 and 28.6% (95% CI: 0, 57.1) for the other influenza viruses. Age and gender were not significantly associated with the proportion of hyperthermia cases among the total of 16 confirmed influenza cases using all three cut-off levels (p > 0.05 for all cut-off levels). Medications were also not associated with hyperthermia among the 16 cases, when the antibiotics were both included and excluded (p > 0.05 for all cut-off levels). Among the 9 cases with H1N1-2009, medications were not significantly associated with hyperthermia (p > 0.05 for all cut-off levels), but the proportion of hyperthermia cases was smaller among those with medications for the cut-off levels of 37.5°C and 38.0°C. For both cut-off levels, the sensitivities of fever for detecting influenza were 16.7% (95% CI: 3.0, 56.4) and 33.3% (95% CI: 6.1, 79.2) among those with and without medications (including antibiotics), respectively.

### Fever among screened passengers

**Figure 4:**
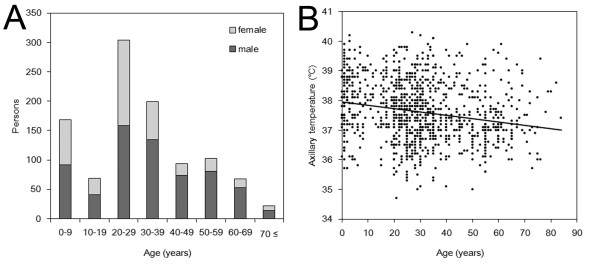
**Age distribution and correlation of age with axillary temperature among the screened passengers (n = 1,049)**. (A) Age distribution of the screened passengers from 1 September 2009 to 31 January 2010. The screened passengers represent those who fulfilled one of the following selection criteria: (a) those who self-reported some symptom or actively visited the health consultation room of the quarantine station; (b) relatives or friends of self-reporting individuals; or (c) those who were detected by an infrared thermoscanner (based on a predefined threshold reading of 35.4°C). (B) Scatter plot of the axillary temperatures as a function of the age of the screened passengers. The straight line is a fitted line by means of a least squares regression (prediction = 37.9-0.011*x*, where *x *is the passenger age). The adjusted coefficient of determination, R^2^, is 0.038.

The age distribution of the 1,049 subjects is shown in Figure 4A. The mean (SD) and median (lower to upper quartiles) ages were 30.3 (18.5) and 29 (20-42) years, respectively. Males accounted for 653 persons (62.7%). Regarding the place of embarkation, 788 cases (75.1%) were from countries belonging to Western Pacific or Southeast Asian regions, 144 (13.7%) were from the Americas, 83 (7.9%) were from Europe and 34 (3.3%) were from the Eastern Mediterranean region, Africa or unknown. A total of 285 persons (27.2%) self-reported some symptoms, and 930 persons (88.7%) were detected by an infrared thermoscanner (Figure 1). Self-reporting individuals with positive screening results by an infrared thermoscanner accounted for 185 cases (64.9% of all self-reporting individuals). Alliances A, B and C accounted for 574, 362 and 113 passengers, respectively.

The mean (SD) axillary temperature was 37.6°C (1.0°C). The proportions of cases with hyperthermia using the cut-off levels of 37.5°C, 38.0°C and 38.5°C were 51.9% (95% CI: 48.8, 54.9), 37.0% (95% CI: 34.1, 40.0) and 23.5% (95% CI: 21.1, 26.2), respectively. The mean (SD) temperature measured by the infrared thermoscanners was 36.3°C (0.9°C). During the period of observation, no confirmatory diagnoses of H1N1-2009 were made among the total screened passengers (i.e. including passengers who were not included in our study). Overall, 28 persons were diagnosed with malaria, and 30 and 15 were diagnosed with dengue virus infection and chikungunya virus infection, respectively.

The axillary temperature tended to be higher among younger passengers (Pearson's correlation coefficient = -0.198, p < 0.01; Figure 4B). Using the cut-off levels of 37.5°C, 38.0°C and 38.5°C, the ages of the passengers with hyperthermia appeared to be significantly younger than those without fever (p < 0.01 for all cut-off levels). There was no gender-specificity in the proportions of hyperthermia for the cut-off levels of 38.0°C and 38.5°C (p = 0.08 and p = 0.15, respectively), whereas gender-specificity was observed for the cut-off level of 37.5°C (p = 0.005; odds ratio of being male with hyperthermia = 0.7 (95% CI: 0.5, 0.9)). Place of embarkation was not significantly associated with hyperthermia (p > 0.05 for all cut-off levels). Self-reporting was not significantly associated with hyperthermia for the cut-off levels of 38.0°C and 38.5°C (p > 0.05 for both), but was significantly associated for the cut-off level of 37.5°C (p = 0.03; odds ratio of being a self-reporting passenger with hyperthermia = 0.7 (95% CI: 0.6, 1.0)), perhaps reflecting the fact that passengers without self-reporting were more likely to be febrile owing to our selection by employing infrared thermoscanners. Satellite was associated with the proportion of hyperthermia (p < 0.01 for all cut-off levels), but the significant association disappeared after adjustment for age using a multiple logistic regression (data not shown).

### Identification of febrile passengers using infrared thermoscanners

**Figure 5:**
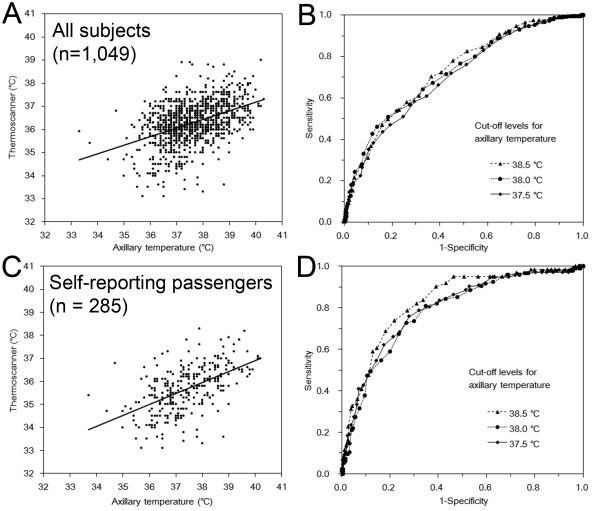
**Relationship between the axillary temperature and the surface temperature measured by an infrared thermoscanner**. (A, C) Scatter plots examining the correlations between the surface temperature measured by an infrared thermoscanner and the axillary temperature. The straight line represents a fitted line by means of a least squares regression. The adjusted coefficients of determination, R^2^, were estimated to be 0.196 and 0.296 for the data shown in (A) and (C), respectively. (B, D) Comparison of the receiver operating characteristic curves showing the relationships between sensitivity (true positives) and 1-specificity (true negatives) in determining the diagnostic performances of the infrared thermoscanners for predicting three different thresholds of hyperthermia definitions (37.5°C, 38.0°C and 38.5°C) based on the axillary temperature. Panels A and B show the data for all the screened passengers (n = 1,049), while panels C and D show the data for the self-reporting passengers only (n = 285).

In a comparison of the axillary temperatures and the surface temperatures measured by the infrared thermoscanners, the Pearson's correlation coefficient was estimated to be 0.44 (p < 0.01). As shown in the scatter plot in Figure 5A, the variances of both measurements were large. Using the three cut-off levels for hyperthermia, the surface temperatures measured by the infrared thermoscanners were significantly higher among those defined as having hyperthermia (p < 0.01 for all cut-off levels). Table 1 shows the diagnostic performances of the infrared thermoscanners in identifying fever at each cut-off level. Using the cut-off levels of 37.5°C, 38.0°C and 38.5°C, the sensitivities were estimated to be 58.3%, 50.8% and 70.4% and the specificities were estimated to be 70.5%, 81.7% and 63.6%, respectively. The PPV and NPV ranged from 37.3-68.0% and 61.1-87.5%, respectively. The PPV was smallest (37.3%) for the cut-off level of 38.5°C, while the NPV was smallest (61.9%) for the cut-off level of 37.5°C. The receiver operating characteristic (ROC) curves for the 1,049 subjects with the three different cut-off levels are shown in Figure 5B. The expected AUC values ranged from 70.5-73.9%, and were much smaller than those in previously published studies [15,18]. Table 1 also summarizes the estimated AUC values after adjustment for age. The adjustment offered only slight improvements, and the age-adjusted AUC ranged from 74.0-75.9%.

**Table 1:** Estimated diagnostic performances of the infrared thermoscanners in predicting hyperthermia measured by the axillary temperature

	Cut-off level of axillary temperature		
**All subjects (n = 1,049)**	**37.5°C**	**38.0°C**	**38.5°C**

Positive fraction^†^	51.9 (48.8, 54.9)	37.0 (34.1, 39.9)	23.5 (21.0, 26.1)
Sensitivity	58.3 (54.1, 62.4)	50.8 (45.8, 55.7)	70.4 (64.8, 76.1)
Specificity	70.5 (66.5, 74.5)	81.7 (78.7, 84.6)	63.6 (60.3, 66.9)
Positive predictive value	68.0 (64.7, 71.3)	61.9 (57.5, 66.4)	37.3 (34.5, 40.2)
Negative predictive value	61.1 (58.3, 63.8)	73.9 (71.8, 75.9)	87.5 (85.3, 89.7)
AUC_unadj_^‡^	70.5 (67.7, 73.2)	72.4 (69.6, 75.0)	73.1 (70.4, 75.7)
AUC_adj_^‡^	74.0 (71.3, 76.6)	75.2 (72.4, 77.7)	75.9 (73.2, 78.4)

Self-reporting (n = 285)			

Positive fraction^†^	46.3 (43.3, 49.3)	46.3 (43.3, 49.3)	21.4 (18.9, 23.9)
Sensitivity	75.8 (68.4, 83.1)	81.1 (74.4, 87.7)	73.8 (62.7, 84.8)
Specificity	71.9 (64.8, 79.0)	65.3 (57.7, 72.8)	78.1 (72.7, 83.5)
Positive predictive value	69.9 (64.2, 75.6	66.8 (61.7, 72.0)	47.9 (40.7, 55.1)
Negative predictive value	77.5 (71.9, 83.0)	80.0 (74.0, 85.9)	91.6 (88.3, 94.9)
AUC_unadj_^‡^	79.5 (74.4, 83.7)	78.3 (73.1, 82.7)	82.9 (78.1, 86.8)
AUC_adj_^‡^	78.8 (73.7, 83.1)	75.0 (69.7, 79.7)	74.0 (68.6, 78.7)

All the values are expressed as percentages. ^†^Proportion of subjects whose axillary temperature was above the specified cut-off level; ^‡^AUC_unadj_, area under the receiver operating characteristic curve without adjustment for age; AUC_adj_, age-adjusted estimate of the area under the receiver operating characteristic curve. Values in parentheses are the 95% confidence intervals.

We also measured the diagnostic performances of the infrared thermoscanners in correctly detecting fever among only the self-reporting passengers (n = 285). A scatter plot is shown in Figure 5C, and the Pearson's correlation coefficient was estimated to be 0.54 (p < 0.01), which was slightly greater than that for the total screened subjects. The estimated sensitivity and PPV were also higher than those of the total screened subjects (ranging from 73.8-81.1% and 47.9-69.9%, respectively), whereas the specificity and NPV were only partially significantly different compared with those for the total screened passengers (Table 1). The ROC curves for the 285 self-reporting passengers are shown in Figure 5D. Despite small improvements, the expected AUC values were as low as 79.5-82.9%. Although the passengers defined as having hyperthermia were significantly younger among the self-reporting passengers (p = 0.003, p = 0.026 and p = 0.004 for the cut-off levels of 37.5°C, 38.0°C and 38.5°C, respectively), adjustment for age did not result in apparent improvement of the estimated AUC (74.0-78.8%).

## Discussion

The present study analyzed epidemiological datasets of confirmed influenza cases whose diagnosis took place at Narita International Airport during the early stages of the 2009 pandemic and of a selected and suspected fraction of passengers screened from September 2009 to January 2010. In our retrospective assessments of the diagnostic performances of fever screening in detecting and diagnosing influenza at the main entrance airport to Japan, three key findings appeared to be notable. First, despite the small sample size, the sensitivity of fever (e.g. 38.0°C) for detecting H1N1-2009 upon arrival was estimated to be as low as 22.2% among the confirmed cases with H1N1-2009. In addition, 5 of the 9 confirmed cases with H1N1-2009 (55.6%) were under antipyretic medications upon arrival. Second, the estimates of the diagnostic performances of the infrared thermoscanners in identifying fever among the selected and suspected fraction of passengers were smaller than those in previously published studies, in which the samples were mostly general populations based on prospective study designs and/or under ideal study conditions [15,16,21,22,27]. For example, the sensitivity and AUC for the cut-off level of 38.0°C in the present study were as low as 50.8% and 72.4%, respectively. Third, even though we examined a suspected fraction of passengers as our subjects (i.e. those who were theoretically more likely to be febrile than the remaining passengers), the PPV still appeared to be as low as 37.3-68.0%. Considering the total passengers arriving at Narita International Airport, the actual PPV will be smaller than our estimates (owing to the smaller prevalence of hyperthermia), implying more false-positive passengers during mass screening if one relies on infrared thermoscanners for active detection of hyperthermia [21]. In summary, our retrospective study demonstrates that reliance on fever alone is unlikely to be feasible as an entry screening measure.

The most important caveat of the present study is that there are two independent processes when considering the diagnostic performances of fever screening at an international border [28]. The first is the sensitivity of fever for detecting influenza cases. Although influenza-like illness (e.g. defined as a temperature higher than 37.8°C plus either cough or sore throat) can be accurately found by clinical examinations, it is known that the clinical findings do not permit the confirmation or exclusion of the diagnosis of influenza [29,30]. Whereas the sensitivity of fever alone is undoubtedly higher than that of influenza-like illness and fever screening may be useful for avoiding a substantial number of false-negatives [31], more critical studies on influenza-like illness have indicated that a high temperature (37.8°C or higher) is not the prime indicator of influenza [32,33]. Thus, even with these facts alone, it is evident that active identification of influenza cases by fever screening alone is unlikely to be feasible. In addition, our experience at Narita International Airport led us to realize that the axillary temperature tends to be readily modified by commercial medications (e.g. antipyretics) in practical settings. Although the proportion of febrile cases among confirmed H1N1-2009 cases was reported to be 94% in the United States [34], no direct comparison can strictly be made because the fraction of febrile cases at an international border is different from that among a total number of confirmed cases in a community. However, that figure of 94% and the figure of 22.2% obtained in our study indicate that the antipyretic medications taken by our study participants potentially reduced the risk of fever by 76.4%.

Second, even though the diagnostic performances of the infrared thermoscanners in detecting fever were not sufficiently high, the prevalence of hyperthermia would be very small among the total number of international passengers, and thus the PPV would be considerably lowered [20,21]. The finding our study adds to the literature on this point is that the PPV of infrared thermoscanners was still insufficient for actively detecting febrile passengers, even when our interest was restricted to a suspected fraction of passengers. The sensitivity of entry screening in correctly detecting and diagnosing symptomatic influenza is measured by the product of the above-mentioned two different sensitivities [28], i.e. the sensitivity of fever for detecting influenza cases and the sensitivity of a non-invasive device for detecting febrile passengers. The PPV of entry screening is therefore smaller than that of the infrared thermoscanners alone. Of course, a confirmatory diagnosis of influenza is further required to account for the limited sensitivity of the rapid diagnostic testing. The present study does not criticize the use of infrared thermoscanners, but does emphasize that reliance on its use during the entry screening of influenza is unlikely to be feasible. Such devices could be used for other purposes (e.g. estimation of true prevalence based on known estimates of sensitivity and specificity among the total passengers) or in other settings (e.g. screening of fever in a setting with a far greater prevalence of hyperthermia), because infrared thermoscanners improve the detection of fever and are especially useful in settings where the PPV and NPV do not matter [35].

Our estimates of the diagnostic performances must be interpreted with caution (Table 1). The analyses of our second dataset were based on a retrospective non-random sample that was considered to represent a suspected fraction of passengers. In other words, the estimated sensitivity and specificity are not applicable to other passengers owing to the imposed selection criteria, and instead are only useful for the sample population that we examined. Nevertheless, given the previous reports of the sensitivity and specificity among a wider spectrum of the population [20,21,27], this point should not be regarded as a negative aspect. The scientific value of our retrospective study was to demonstrate that the diagnostic performances of infrared thermoscanners in detecting febrile passengers, especially the sensitivity, can be even worse among the suspected fraction of passengers than among all the passengers. In addition to previous studies indicating that the use of infrared thermoscanners for fever screening prior to voluntary self-reporting was not fully justified [20-22], our study has demonstrated that infrared thermoscanners were not useful for actively detecting fever, even among a selected and suspected fraction of passengers. Our investigation of a selected and suspected fraction of passengers only, especially with the inclusion of those detected by the infrared thermoscanners, could partly provide a reason for the small estimates of the specificity. For example, owing to the representation of the suspected fraction of passengers, there were not many subjects with low axillary temperatures among our subjects, thereby leading to small estimates of the specificity compared with all arriving passengers. Since the inclusion of cases detected by the infrared thermoscanners in our samples complicates an explicit interpretation of our estimates, we also examined the diagnostic performances only among the self-reported cases. The estimates of PPV and NPV among the self-reporting passengers did not differ significantly from those among our total subjects.

In addition to the limited diagnostic performance of fever screening in identifying febrile influenza cases, it should be remembered that the readings of infrared thermoscanners are known to be influenced by other confounding factors, most notably by age and outdoor temperature [15,20-22]. Although we were not able to adjust for room temperature owing to its variation depending on air-conditioning and individual routes (e.g. gate and satellite combinations), age was shown to be a confounding factor, even among the suspected fraction of passengers. There are two plausible explanations for these findings: (a) physiological reasons including age-dependent vascular reactivity (e.g. the temperature varies more easily among children than among elderly persons) [36] and (b) influenza H1N1-2009 has mainly been observed in younger individuals, most notably among school-age children [37-40]. Although no confirmatory diagnoses of H1N1-2009 were made during the screening from September 2009 to January 2010, it is likely that substantial numbers of undetected cases were allowed into Japan during the study period [41]. The above-mentioned point (b) poses a technical challenge, because the real-time dependence of age on the epidemiology of influenza introduces a time-dependency in its influence on the readings of the infrared thermoscanners (i.e. a simple statistical adjustment does not hold in such instances). As an additional complication but perhaps one of the most important features among international passengers, our experience at Narita International Airport led us to realize that the use of antipyretics and antivirals is very likely among febrile passengers in practical settings, thereby greatly complicating the detection owing to masked symptoms. Among those with any suspicious symptoms, it is natural that medications with commercially available antipyretics are widely used without any restrictions, and the different timings, doses and medicines do not permit us to adjust for the influence by statistical modeling.

Except for cases of imminent public health risk, the revised International Health Regulations (IHR) in 2005 were intended to minimize interference with world travel, permitting only non-invasive and least intrusive medical examinations that could achieve a "public health objective" [42]. Although infrared thermoscanners are non-invasive and may detect a small portion of febrile influenza cases among the total passengers, our study has demonstrated fundamental problems in the reliance on fever in detecting and diagnosing influenza in international passengers. In addition to the issue of screening, the effectiveness of entry screening involves the presence of incubating individuals [5,43] and asymptomatic cases [7,8]. Given the limited information that we can gain from fever alone, one could further examine other vital signs to improve the detection during mass screening [44], along with efforts to promote self-reporting and improve its coverage. In addition to such devices, it is vital to reconsider the public health objectives of entry screening measures with a specific disease in mind (e.g. influenza) [45], and the way forward requires us to explicitly define the roles and purposes of international border control in the event of the next pandemic [46].

## Conclusions

To retrospectively assess the feasibility of detecting the cases of influenza (H1N1-2009) based on fever screening as a sole measure in a practical setting, we analyzed epidemiological datasets of confirmed influenza cases whose diagnosis took place at Narita International Airport during the early stages of the 2009 pandemic and of a selected and suspected fraction of passengers screened from September 2009 to January 2010. Among the confirmed H1N1-2009 cases (n = 9), the sensitivity of fever for detecting influenza upon arrival appeared to be as low as 22.2%, and 5 of the 9 cases (55.6%) were under antipyretic medications. The PPV of the infrared thermoscanners for detecting fever among the suspected fraction of passengers (n = 1,049) was shown to be insufficient to actively detect febrile influenza cases among passengers. Given the additional presence of confounding factors and unrestricted medications among passengers, the reliance on fever alone is unlikely to be feasible as an entry screening measure against influenza.

## Competing interests

The authors declare that they have no competing interests.

## Authors' contributions

KK and HN conceived the study. HN developed the methodological ideas and implemented the statistical analyses. HN drafted the manuscript and all the authors discussed and revised the manuscript. All authors read and approved the final manuscript.

## Pre-publication history

The pre-publication history for this paper can be accessed here:

http://www.biomedcentral.com/1471-2334/11/111/prepub

## References

[B1] Bell DM World Health Organization Working Group on International and Community Transmission of SARS Public health interventions and SARS spread, 2003 Emerg Infect Dis 2004 10 1900 6 15550198 10.3201/eid1011.040729 PMC3329045

[B2] St John RK King A de Jong D Bodie-Collins M Squires SG Tam TW Border screening for SARS Emerg Infect Dis 2005 11 6 10 15705315 10.3201/eid1101.040835 PMC3294328

[B3] Bell DM World Health Organization Writing Group Non-pharmaceutical interventions for pandemic influenza, international measures Emerg Infect Dis 2006 12 81 7 16494722 10.3201/eid1201.051370 PMC3291414

[B4] McLeod MA Baker M Wilson N Kelly H Kiedrzynski T Kool JL Protective effect of maritime quarantine in South Pacific jurisdictions, 1918-19 influenza pandemic Emerg Infect Dis 2008 14 468 70 10.3201/eid1403.070927 18325264 PMC2570822

[B5] Pitman RJ Cooper BS Trotter CL Gay NJ Edmunds WJ Entry screening for severe acute respiratory syndrome (SARS) or influenza: policy evaluation BMJ 2005 331 1242 3 16176938 10.1136/bmj.38573.696100.3A PMC1289362

[B6] Glass K Becker NG Evaluation of measures to reduce international spread of SARS Epidemiol Infect 2006 134 1092 101 10.1017/S0950268806005863 16476169 PMC2870475

[B7] Carrat F Vergu E Ferguson NM Lemaitre M Cauchemez S Leach S Valleron AJ Time lines of infection and disease in human influenza: a review of volunteer challenge studies Am J Epdiemiol 2008 167 775 85 10.1093/aje/kwm375 18230677

[B8] Nishiura H Wilson N Baker MG Quarantine for pandemic influenza control at the borders of small island nations BMC Infect Dis 2009 9 27 10.1186/1471-2334-9-27 19284571 PMC2670846

[B9] Inaba H Nishiura H The state-reproduction number for a multistate class age structured epidemic system and its application to the asymptomatic transmission model Math Biosci 2008 216 77 89 10.1016/j.mbs.2008.08.005 18768142

[B10] Cowling BJ Lau LL Wu P Wong HW Fang VJ Riley S Nishiura H Entry screening to delay local transmission of 2009 pandemic influenza A (H1N1) BMC Infect Dis 2010 10 82 10.1186/1471-2334-10-82 20353566 PMC3152767

[B11] Governmental Committee of Action Plans against Influenza Pandemic Action Plans against Influenza Pandemic 2009 Tokyo, The Government of Japan http://www.cas.go.jp/jp/seisaku/ful/kettei/071026keikaku.pdf in Japanese [last accessed on 16 November 2010]

[B12] Chan LS Cheung GT Lauder IJ Kumana CR Lauder IJ Screening for fever by remote-sensing infrared thermographic camera J Travel Med 2004 11 273 9 15544710 10.2310/7060.2004.19102

[B13] Hughes WT Patterson GG Thornton D Williams BJ Lott L Dodge R Detection of fever with infrared thermometry: a feasibility study J Infect Dis 1985 152 301 6 10.1093/infdis/152.2.301 4031545

[B14] McBride WJ Buikstra E FitzGerald M Investigation of febrile passengers detected by infrared thermal scanning at an international airport Aust NZ J Public Health 2010 34 5 10 10.1111/j.1753-6405.2010.00466.x 20920098

[B15] Ng EY Kaw GJ Chang WM Analysis of IR thermal imager for mass blind fever screening Microvasc Res 2004 68 104 9 10.1016/j.mvr.2004.05.003 15313119

[B16] Ng EY Is thermal scanner losing its bite in mass screening of fever due to SARS? Med Phys 2005 32 93 7 10.1118/1.1819532 15719959 PMC7168465

[B17] Ng DK Chan CH Lee RS Leung LC Non-contact infrared thermometry temperature measurement for screening fever in children Ann Trop Paediatr 2005 25 267 75 10.1179/146532805X72412 16297301

[B18] Chiu WT Lin PW Chiou HY Lee WS Lee CN Yang YY Lee HM Hsieh MS Hu CJ Ho YS Deng WP Hsu CY Infrared thermography to mass-screen suspected SARS patients with fever Asia Pac J Public Health 2005 17 26 8 10.1177/101053950501700107 16044829

[B19] Liu CC Chang RE Change WC Limitations of forehead infrared body temperature detection for fever screening for severe acute respiratory syndrome Infect Control Hosp Epidemiol 2004 25 1109 11 10.1086/502351 15636300

[B20] Bitar D Goubar A Desenclos JC International travels and fever screening during epidemics: a literature review on the effectiveness and potential use of non-contact infrared thermometers Euro Surveill 2009 14 pii19115 19215720

[B21] Hausfater P Zhao Y Defrenne S Bonnet P Riou B Cutaneous infrared thermometry for detecting febrile patients Emerg Infect Dis 2008 14 1255 8 18680649 10.3201/eid1408.080059 PMC2600390

[B22] Suzuki T Wada K Wada Y Kagitani H Arioka T Maeda K Kida K The validity of mass body temperature screening with ear thermometers in a warm thermal environment Tohoku J Exp Med 2010 222 89 95 10.1620/tjem.222.89 20877164

[B23] Shimada T Gu Y Kamiya H Komiya N Odaira F Sunagawa T Takahashi H Toyokawa T Tsuchihashi Y Yasui Y Tada Y Okabe N Epidemiology of influenza A(H1N1)v virus infection in Japan, May-June 2009 Euro Surveill 2009 14 pii = 19244 19555600 10.2807/ese.14.24.19244-en

[B24] Department of Quarantine, Narita Airport Quarantine Station Report of responses to pandemic influenza at the Narita Airport Quarantine Station 2009 Chiba, Narita Airport Quarantine Station http://www.forth.go.jp/keneki/narita/pdf/20090805_NQ_H1N1%20flu_Report.pdf in Japanese [last accessed on 16 Novmber 2010]

[B25] Mercaldo ND Lau KF Zhou XH Confidence intervals for predictive values with an emphasis to case-control studies Stat Med 2007 26 2170 83 10.1002/sim.2677 16927452

[B26] Gengsheng Qin Hotilovac L Comparison of non-parametric confidence intervals for the area under the ROC curve of a continuous-scale diagnostic test Stat Methods Med Res 2008 17 207 21 18426855 10.1177/0962280207087173

[B27] Nguyen AV Cohen NJ Lipman H Brown CM Molinari NA Jackson WL Kirking H Szymanowski P Wilson TW Salhi BA Roberts RR Stryker DW Fishbein DB Comparison of 3 infrared thermal detection systems and self-report for mass fever screening Emerg Infect Dis 2010 16 1710 7 21029528 10.3201/eid1611.100703 PMC3294528

[B28] Nishiura H Joint quantification of transmission dynamics and diagnostic accuracy applied to influenza Math Biosc Eng 2011 8 49 64 10.3934/mbe.2011.8.49 21361399

[B29] Call SA Vollenweider MA Hornung CA Simel DL McKinney WP Does this patient have influenza? JAMA 2005 293 987 97 10.1001/jama.293.8.987 15728170

[B30] Nishiura H Real-time forecasting of an epidemic using a discrete time stochastic model: a case study of pandemic influenza (H1N1-2009) Biomed Eng Online 2011 10 15 10.1186/1475-925X-10-15 21324153 PMC3045989

[B31] Kasper MR Wierzba TF Sovann L Blair PJ Putnam SD Evaluation of an influenza-like illness case definition in the diagnosis of influenza among patients with acute febrile illness in Cambodia BMC Infect Dis 2010 10 320 10.1186/1471-2334-10-320 21054897 PMC2988054

[B32] Thursky K Cordova SP Smith D Kelly H Working towards a simple case definition for influenza surveillance J Clin Virol 2003 27 170 9 10.1016/S1386-6532(02)00172-5 12829039

[B33] Babcock HM Merz LR Fraser VJ Is influenza an influenza-like illness? Clinical presentation of influenza in hospitalized patients Infect Control Hosp Epidemiol 2006 27 266 70 10.1086/501539 16532414

[B34] Novel Swine-Origin Influenza A (H1N1) Virus Investigation Team Dawood FS Jain S Finelli L Shaw MW Lindstrom S Garten RJ Gubareva LV Xu X Bridges CB Uyeki TM Emergence of a novel swine-origin influenza A (H1N1) virus in humans N Engl J Med 2009 360 2605 15 19423869 10.1056/NEJMoa0903810

[B35] Priest PC Duncan AR Jennings LC Baker MG Thermal image scanning for influenza border screening: Results of an airport screening study PLoS One 2011 6 e14490 10.1371/journal.pone.0014490 21245928 PMC3016318

[B36] Andersson SE Edvinsson ML Edvinsson L Cutaneous vascular reactivity is reduced in aging and in heart failure: association with inflammation Clin Sci 2003 105 699 707 10.1042/CS20030037 12848618

[B37] Nishiura H Travel and age of influenza A (H1N1) 2009 virus infection J Travel Med 2010 17 269 70 10.1111/j.1708-8305.2010.00418.x 20636601

[B38] Reichert T Chowell G Nishiura H Christensen RA McCullers JA Does Glycosylation as a modifier of Original Antigenic Sin explain the case age distribution and unusual toxicity in pandemic novel H1N1 influenza? BMC Infect Dis 2010 10 5 10.1186/1471-2334-10-5 20059763 PMC3003248

[B39] Wada K Nishiura H Kawana A An epidemiological analysis of severe cases of the influenza A (H1N1) 2009 virus infection in Japan Influenza Other Respi Viruses 2010 4 179 86 10.1111/j.1750-2659.2010.00143.x 20836793 PMC5964544

[B40] Nishiura H Cook AR Cowling BJ Assortativity and the probability of epidemic extinction: A case study of pandemic influenza A (H1N1-2009) Interdiscip Perspect Infect Dis 2011 2011 194507 http://www.hindawi.com/journals/ipid/2011/194507.html 21234337 10.1155/2011/194507 PMC3017939

[B41] Nishiura H Chowell G Castillo-Chavez C Did modeling overestimate the transmission potential of pandemic (H1N1-2009)? Sample size estimation for post-epidemic seroepidemiological studies PLoS One 2011 6 e17908 10.1371/journal.pone.0017908 21455307 PMC3063792

[B42] World Health Organization International Health Regulations (2005) 2005 Geneva, World Health Organization http://www.who.int/ihr/en/[last accessed on 16 Novmber 2010]

[B43] Nishiura H Inaba H Estimation of the incubation period of influenza A (H1N1-2009) among imported cases: Addressing censoring using outbreak data at the origin of importation J Theor Biol 2011 272 123 30 10.1016/j.jtbi.2010.12.017 21168422

[B44] Matsui T Hakozaki Y Suzuki S Usui T Kato T Hasegawa K Sugiyama Y Sugamata M Abe S A novel screening method for influenza patients using a newly developed non-contact screening system J Infect 2010 60 271 7 10.1016/j.jinf.2010.01.005 20138082 PMC7112665

[B45] Omori R Nishiura H Theoretical basis to measure the impact of short-lasting control of an infectious disease on the epidemic peak Theor Biol Med Model 2011 8 2 10.1186/1742-4682-8-2 21269441 PMC3040699

[B46] Cooper BS Pitman RJ Edmunds WJ Gay NJ Delaying the international spread of pandemic influenza PLoS Med 2006 3 e212 10.1371/journal.pmed.0030212 16640458 PMC1450020

